# What does engagement mean to you? Exploring the experiences of the League older adult advisory group for social robot technology research

**DOI:** 10.1002/alz.092681

**Published:** 2025-01-09

**Authors:** Susanna E. Martin, Cindy C. Zhang, Julie M. Robillard

**Affiliations:** ^1^ University of British Columbia, Vancouver, BC Canada; ^2^ BC Children’s and Women’s Hospital, Vancouver, BC Canada

## Abstract

**Background:**

Co‐creation methods are increasingly being used in the research and development of technologies that support older adults living with dementia and their care partners to live well. Use of collaborative methods to engage with the dementia community helps to ensure that research processes and end solutions are sensitively designed, reflective of needs and values, and responsive to priorities. Engagement also has proximal benefits for older adults: Being involved in purposeful activity has been shown to positively impact health and wellbeing outcomes. However, despite these benefits, the value of co‐creation methods in dementia research remains under‐investigated from the perspectives of older adults themselves. Here we seek to address this gap in the context of social robot research exploring the impact and value that older adults derive from engaging in dementia technology research.

**Method:**

We carry out semi‐structed interviews with older adults who are members of a lived experience advisory group, known as the League. League members provide ongoing consultation on research activities carried out at the Neuroscience, Engagement and Smart Tech Lab.

League members also complete surveys, including a subset of questions from the Patient Engagement In Research (PEIR) Framework, to capture quantitative responses on key components of engagement. We adopt a social‐technical perspective to generate a holistic appreciation for the impacts of engagement.

**Result:**

Individual perspectives captured in this mixed methods study reveal that older adults have unique experiences with technology, and technology research, that modulate their level of engagement and the meanings that they derive from participation. Results demonstrate the impact that engagement has on biopsychosocial factors, roles, and identity, and underscore the ethical imperative of human‐centered research methods.

**Conclusion:**

Insights will support the evidence base for person‐centred, co‐creation methodologies in dementia research, and showcase the potential for meaningful engagement to support the health and wellbeing of older adult collaborators.

